# Connexin 43 hemichannels and prostaglandin E_2_ release in anabolic function of the skeletal tissue to mechanical stimulation

**DOI:** 10.3389/fcell.2023.1151838

**Published:** 2023-04-13

**Authors:** Dezhi Zhao, Jiawei Wu, Francisca M. Acosta, Huiyun Xu, Jean X. Jiang

**Affiliations:** ^1^ School of Medicine, Northwest University, Xi’an, China; ^2^ Department of Biochemistry and Structural Biology, University of Texas Health Science Center, San Antonio, TX, United States; ^3^ School of Life Sciences, Northwestern Polytechnical University, Xi’an, China

**Keywords:** connexin 43, hemichannel, prostaglandin E2, bone, mechanical stimulation

## Abstract

Bone adapts to changes in the physical environment by modulating remodeling through bone resorption and formation to maintain optimal bone mass. As the most abundant connexin subtype in bone tissue, connexin 43 (Cx43)-forming hemichannels are highly responsive to mechanical stimulation by permitting the exchange of small molecules (<1.2 kDa) between bone cells and the extracellular environment. Upon mechanical stimulation, Cx43 hemichannels facilitate the release of prostaglandins E_2_ (PGE_2_), a vital bone anabolic factor from osteocytes. Although most bone cells are involved in mechanosensing, osteocytes are the principal mechanosensitive cells, and PGE_2_ biosynthesis is greatly enhanced by mechanical stimulation. Mechanical stimulation-induced PGE_2_ released from osteocytic Cx43 hemichannels acts as autocrine effects that promote *β*-catenin nuclear accumulation, Cx43 expression, gap junction function, and protects osteocytes against glucocorticoid-induced osteoporosis in cultured osteocytes. *In vivo*, Cx43 hemichannels with PGE_2_ release promote bone formation and anabolism in response to mechanical loading. This review summarizes current *in vitro* and *in vivo* understanding of Cx43 hemichannels and extracellular PGE_2_ release, and their roles in bone function and mechanical responses. Cx43 hemichannels could be a significant potential new therapeutic target for treating bone loss and osteoporosis.

## Introduction

Bone is a mechanosensitive tissue that undergoes constant remodeling to adapt to the physical environment (Bonewald, 2011). Enhanced mechanical stimulation has major, positive anabolic impacts on bone tissue (Warden et al., 2007; Erlandson et al., 2012), whereas disuse leads to bone loss (Lang et al., 2004). As the most abundant and long-lived cells in the adult skeleton (Bonewald, 2011), osteocytes with extensive lacunar-canalicular networks are generally regarded as mechanosensory cells that help translate mechanical stimulation into biological signals by regulating the function of osteoclasts and osteoblasts on the bone surface. Prostaglandin E_2_ (PGE_2_), a member of the eicosanoid family, is an essential key factor involved in the anabolic response of bone tissue to mechanical loading. PGE_2_ is not stored by bone cells but is synthesized in response to mechanical stimulation (Klein-Nulend et al., 1997). PGE_2_ at low concentrations (0–1 nM) stimulates osteoblast proliferation and differentiation, whereas at high concentrations (≥1 nM) inhibits osteogenesis (Ozawa et al., 1990). Mechanical loading induces the expression of cyclooxygenase 2 (COX-2), a key enzyme for PGE_2_ synthesis (Blackwell et al., 2010), and intracellular PGE_2_ is released under mechanical stimulation (Ajubi et al., 1996; Ajubi et al., 1999). *In vitro* studies show that released PGE_2_ from mechanically stimulated osteocytes can reduce *SOST*/sclerostin expression through EP4 receptors (Galea et al., 2011) and also enhance osteoclast activity (Chan et al., 2009; Matsuzaka et al., 2021). Besides osteocytes, osteoblasts also release PGE_2_ in response to mechanical stimulation (Duncan and Turner, 1995; Klein-Nulend et al., 1997; Saunders et al., 2001), which influences osteoblast proliferation and differentiation (Imamura et al., 1990; Miwa et al., 1991). *In vivo* studies also show that mechanical loading increases PGE_2_ levels in the tibia bone of humans (Thorsen et al., 1996) and mice (Zhao et al., 2022a; Zhao et al., 2022b). An earlier study reported that intermittent PGE_2_ treatment increases bone formation and bone mass (Jee et al., 1985; Tian et al., 2007), whereas, inhibition of PGE_2_ suppresses bone formation induced by mechanical loading (Forwood, 1996).

One form of cell-cell communication is via gap junctions, the membrane-spanning channels composed of two juxtaposed hemichannels (Goodenough et al., 1996). In addition to direct gap junction intercellular communication (GJIC), halves of gap junctions, hemichannels mediate the communication between bone cells and the extracellular environment (Civitelli, 2008). Connexin-forming hemichannels exhibit relatively low substrate selectivity and permit small molecules (≤1.2 kDa) to pass through (Goodenough et al., 1996; Kumar and Gilula, 1996). Cx43 is the predominant connexin subtype expressed in osteocytes (Yellowley et al., 2000; Cheng et al., 2001a) and osteoblasts (Civitelli et al., 1993). Cx43 hemichannels are highly responsive to mechanical stimulation, and their opening induced by mechanical loading mediates the release of anabolic factors such as PGE_2_, adenosine triphosphate (ATP) and nitric oxide (NO) from osteocytes (Jiang and Cherian, 2003; Cherian et al., 2005) and osteoblasts (Thi et al., 2012). Both ATP and NO are related to the production of the bone anabolic agent PGE_2_ (Sugiatno et al., 2006; Genetos et al., 2007). In osteocytes, released PGE_2_ can act in either a feed-forward or feedback manner in regulating Cx43 expression and function. Mechanical loading-induced PGE_2_ release from osteocytic Cx43 hemichannels increases Cx43 expression and Cx43-forming gap junctions (Cheng et al., 2001b). Interestingly, fluid flow-induced accumulation of extracellular PGE_2_ leads to the closure of Cx43 hemichannels (Riquelme et al., 2015). The Cx43 hemichannels and extracellular PGE_2_ play an inhibitory role in glucocorticoid-induced apoptosis (Kitase et al., 2010). Cx43 hemichannels with PGE_2_ release are also essential for normal bone structure (Xu et al., 2015; Zhao et al., 2022a) and the anabolic response of tibias to mechanical loading *in vivo* (Zhao et al., 2022a; Zhao et al., 2022b). In addition to connexins, pannexins are also capable of forming hemichannels (Plotkin et al., 2017). Pannexin1 is the most widely distributed pannexin in bone cells, and these hemichannels are involved in the release of PGE_2_ induced by mechanical stimulation in osteoblasts (Thi et al., 2012). However, the roles of pannexin channels in bone have not been investigated in great detail. In this review, we focus on the function of Cx43 hemichannels in releasing PGE_2_, and further PGE_2_-regulated skeletal development and cellular signals that drive bone anabolic and bone remodeling responses to mechanical stimulation.

### Relationship between Cx43 hemichannels and PGE_2_ upon mechanical stimulation *in* osteocytes

Osteocytes are a rich source of PGE_2_ upon mechanical stimulation. Mechanical stress in the form of fluid flow causes a rapid increase in COX-2 expression (Joldersma et al., 2000) and PGE_2_ production in osteocytes (Ajubi et al., 1996; Ajubi et al., 1999). Inhibition of COX-2 enzymatic activity with NS-398 inhibitor abolishes the stimulatory effect of fluid flow on PGE_2_ secretion from osteocytes (Bakker et al., 2003). During mechanical stimulation, Cx43 hemichannels play a crucial role in the PGE_2_ release from osteocytes. Low-density cultures of primary osteocytes and osteocyte-like MLO-Y4 cells with minimal cell-cell contacts, thus void of gap junctions, release more PGE_2_ than cells cultured at higher densities (Jiang and Cherian, 2003; Cherian et al., 2005). Inhibition of Cx43 channels by chemical blocker *β*-glycyrrhetinic acid (Jiang and Cherian, 2003; Cherian et al., 2005) or knocking down Cx43 by siRNA (Genetos et al., 2007) attenuates fluid flow-induced hemichannel activity and PGE_2_ production in low-density cultured osteocytes. This experimental evidence suggests that Cx43 hemichannels participate in PGE_2_ secretion during mechanical stimulation. To further depict the relationship between Cx43 hemichannels and PGE_2_ release, we develop a polyclonal antibody, Cx43 (E2), that targets the second extracellular loop domain of Cx43 and specifically blocks osteocytic Cx43 hemichannels *in vitro* (Siller-Jackson et al., 2008; Riquelme et al., 2013). Blocking of Cx43 hemichannels by Cx43 (E2) inhibits the opening of hemichannels and the release of PGE_2_ induced by flow shear stress in osteocytes (Siller-Jackson et al., 2008). Interestingly, PGE_2_ also has a negative feedback regulation on Cx43 hemichannels in response to mechanical stimulation. Extracellular PGE_2_ accumulation after the continuous opening of hemichannels by fluid flow acts on EP2/4 receptors to close Cx43 hemichannels (Riquelme et al., 2015). The negative feedback is caused by the PGE_2_ activation of p44/42 ERK signaling and direct Cx43 phosphorylation at S279/282 residues thereby leading to the closure of Cx43 hemichannels. (Riquelme et al., 2015). In addition, the released PGE_2_ from osteocytes by fluid shear stress promotes Cx43 expression and further increases Cx43 gap junctions (Jiang and Cheng, 2001; Xia et al., 2010). Consistent with the effects of fluid shear stress, direct treatment of the MLO-Y4 cells with PGE_2_ similarly increases Cx43 expression and gap junctions. In contrast, inhibition of PGE_2_ signaling by indomethacin reduced gap junction formation by fluid shear stress (Cheng et al., 2001b).

**FIGURE 1:**
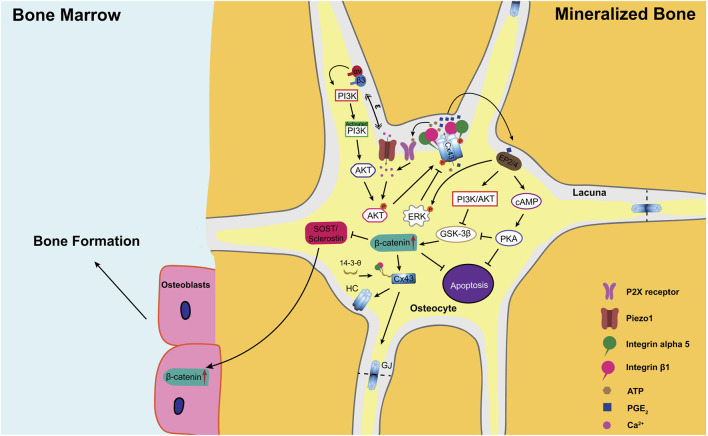
A model illustrating the role of PGE_2_ released from Cx43 hemichannels under mechanical loading in the regulation of the anabolic response to mechanical stimulation in bone. Upon mechanical loading, osteocytic dendrites sense mechanical stimulation and transduce these signals through integrins αvβ3 to activate intracellular PI3K signaling (Wang et al., 2007; Riquelme et al., 2021). In addition, Ca^2+^ influx through Piezo1 also activates PI3K signaling (Zeng et al., 2022). Activated PI3K activates its downstream effector AKT through protein phosphorylation (Batra et al., 2014a). AKT, in turn, directly phosphorylates both Cx43 and integrin alpha 5 (α5) subunit (Batra et al., 2014a), which is required for the interaction between these two proteins (Batra et al., 2012). Additionally, the scaffolding molecule 14-3-3θ facilitates the delivery of Cx43 and integrin a5 from the Golgi apparatus to the plasma membrane to form mechanosensitive Cx43 hemichannels (Batra et al., 2014b). Upon mechanical stimulation, integrin α5β1 is activated and triggers the opening of hemichannels through the conformational change of the integrin (Batra et al., 2012). The opened Cx43 hemichannels mediate the export of intracellular PGE_2,_ whose synthesis is greatly increased by mechanical loading (Cherian et al., 2005; Siller-Jackson et al., 2008). Released PGE_2_ acts in an autocrine/paracrine manner through EP2/EP4 receptors to activate both cAMP/PKA and PI3k/Akt pathways (Xia et al., 2010). These two pathways prevent osteocyte apoptosis and promote nuclear translocation and accumulation of *β*-catenin in osteocytes (Kitase et al., 2010), increasing Cx43 expression and gap junction formation (Xia et al., 2010). In addition, increased *β*-catenin suppresses the sclerostin expression in osteocytes and enhances osteoblast activity and bone formation on endosteal surfaces (Zhao et al., 2022a; Zhao et al., 2022b). Moreover, high extracellular PGE_2_ acts on EP2/4 receptors to activate ERK signaling, which directly phosphorylates Cx43 to promote the closure of the Cx43 hemichannels (Riquelme et al., 2015). GJ, gap junction; HC, hemichannel.

Since the PGE_2_ secretion by osteocytes depends on the opening of Cx43 hemichannels in osteocytes, it is important to understand how Cx43 hemichannels are regulated by mechanical stimulation (Figure 1). We find that the dendritic processes of osteocytes transmit mechanical signals to the cell body, leading to the opening of Cx43 hemichannels in MLO-Y4 cells and primary osteocytes (Burra et al., 2010). Cx43 is richly present in the cell body, not the dendritic processes of osteocytes. The integrin αVβ3, located at the dendrites of osteocytes, is an important component of the glycocalyx complex that tethers osteocytes to the canalicular wall and amplifies the magnitude of mechanical signals experienced by osteocytes (Wang et al., 2007; Riquelme et al., 2021). Upon fluid flow, the force generated by tethering elements is a magnitude higher than shear stress on the cell surface. Mechanically activated integrin αVβ3 at dendritic processes induces the activation of intracellular PI3K signaling (Batra et al., 2012), which activates the downstream effector AKT (Batra et al., 2014a). Activated AKT directly phosphorylates Cx43 and integrin α5 (Batra et al., 2014a) in the cell body to increase the interaction between these two proteins, opening the Cx43 hemichannels. Upon mechanical loading, activation of α5β1 through its conformational changes opens the Cx43 hemichannels in MLO-Y4 cells. Interestingly, this action is independent of integrin binding to its extracellular substrate, fibronectin (Batra et al., 2012; Batra et al., 2014a). Moreover, the scaffolding molecule 14-3-3θ assists in transporting both Cx43 and integrin a5 from the Golgi apparatus to the plasma membrane to form mechanosensitive Cx43 hemichannels (Batra et al., 2014b). Silencing 14-3-3θ prevents the accumulation of Cx43 on the cell membrane and the opening of hemichannels caused by fluid flow (Batra et al., 2014b). Recently, we find that Piezo1 is co-localized with Cx43 hemichannels on osteocyte cell surface. The activation of the Piezo1 leads to an increase in intracellular Ca^2+^ and the opening of Cx43 hemichannels through PI3K-AKT pathway in osteocytes (Zeng et al., 2022). Interestingly, the release of PGE_2_ is upregulated when Piezo1 is activated by either agonist or mechanical stretch (Li et al., 2019; Yang et al., 2022). The PGE_2_ release by Cx43 hemichannels is also regulated by extracellular ATP. Mechanical stimulation-induced ATP release through Cx43 hemichannels activates P2X receptors and promotes the Ca^2+^ influx to sustain the activities of Cx43 hemichannels (Zeng et al., 2022). Blockade of P2X7 purinergic receptors prevents PGE_2_ release from MLO-Y4 cells. However, the activation of purinergic receptors and the increase in the release of PGE_2_ appears to be independent of hemichannel formation (Genetos et al., 2007). Thus, the mechanism for ATP-induced PGE_2_ release is yet to be fully understood in osteocytes.

### PGE_2_, Cx43 hemichannels, and mechanical stimulation in other bone cell types

Besides osteocytes, osteoblast is another mechano-responsive bone cell type (Johnson et al., 1996; Romanello et al., 2001; Liu et al., 2022). Earlier studies report that fluid flow stimulates the release of PGE_2_ from primary osteoblasts and MC3T3-E1 osteoblastic cells (Duncan and Turner, 1995; Klein-Nulend et al., 1997; Saunders et al., 2001). In contrast to osteocytes, osteoblasts require high magnitudes of shear stress for PGE_2_ production (Saunders et al., 2003; Genetos et al., 2005). Lower fluid flow levels induce greater PGE_2_ production in MLO-Y4 osteocyte-like cells than in 2T3 osteoblasts (Kamel et al., 2010). Moreover, Genetos and others even show that fluid flow only activates hemichannels in MLO-Y4, leading to the release of PGE_2_, but not osteoblastic MC3T3-E1 cells (Genetos et al., 2007). Thus, osteoblasts appear to be less mechanically sensitive to PGE_2_ release than osteocytes. Furthermore, increased PGE_2_ level (>10 nM) by hypergravity or compressive pressure promotes proliferation but suppresses differentiation of MC3T3-E1 cells (Ozawa et al., 1990; Miwa et al., 1991).

Interestingly, Cx43 hemichannels expressed in osteoblasts (Romanello and D'Andrea, 2001) can be regulated by mechanical stimulation (Romanello et al., 2003). However, PGE_2_ release appears not to be driven by Cx43 hemichannels in osteoblasts. Cx43-null calvarial osteoblasts still respond to mechanical stimulation, as evidenced by increased dye uptake and PGE_2_ release. In contrast, fluid flow-induced PGE_2_ release is abolished in osteoblasts deficient in pannexin1 (Thi et al., 2012). Pannexin1 is a transmembrane channel with a similar topology as connexins that only form hemichannels but not gap junctions (Penuela et al., 2013). These findings suggest that hemichannels formed by pannexin1 and not Cx43 might be responsible for fluid flow-induced PGE_2_ release in osteoblasts.

Recent studies indicate that osteoclasts are responsive to mechanical stress. Osteoclasts, after mechanical stretch, polarized to the M2 phenotype associated with YAP activation and nuclear translocation, which facilitates osteogenesis of bone marrow-derived mesenchymal stem cells (BMSCs) (Dong et al., 2021). Jiang and others find that the extracellular PGE_2_, acting via EP4 receptors in osteoclasts, activates the Gαs/PI3K/AKT/MAPK signaling pathway and mediates migration and osteoclastogenesis during the progression of osteoarthritis (Jiang et al., 2022). Although it is known that osteoclasts express Cx43 on the plasma membrane and form hemichannels (Vesely et al., 1992; Ilvesaro et al., 2000; Ilvesaro and Tuukkanen, 2003), whether osteoclasts secret PGE_2_ through Cx43 hemichannels remains elusive.

### The signaling pathways activated by PGE_2_ in osteocytes upon mechanical stimulation

PGE_2_ can activate four subtypes of G-protein-coupled receptors (GPCRs), named EP1, EP2, EP3, and EP4 (Woodward et al., 2011). EP2 and EP4 are the most extensively studied in bone (Furuyashiki and Narumiya, 2011). EP2 is a mechanosensitive PGE_2_ receptor whose expression can be enhanced by fluid flow in osteocytes (Cherian et al., 2003). Inhibition of the EP2 receptor by antagonist AH6809 suppresses the production of PGE_2_ and Cx43 expression (Cherian et al., 2003). The fluid flow-induced PGE_2_ release from osteocytes exerts autocrine effects on EP2 receptors to activate the cAMP-PKA pathway in osteocytes and increase the Cx43 gap junction formation (Cherian et al., 2003). Further study reveals that PGE_2_ released from osteocytes by mechanical stimulation could lead to activation of the PI3K/Akt signaling pathway in addition to the cAMP/PKA pathway. The activation of both PI3K/Akt and cAMP/PKA pathways results in the phosphorylation and inactivation of GSK-3β (Xia et al., 2010), which is responsible for the phosphorylation of *β*-catenin, resulting in its ubiquitination and degradation by the 26S proteasome complex (Aberle et al., 1997). Consequently, the inactivated GSK-3β causes an increase in nuclear translocation and accumulation of *β*-catenin in osteocytes (Xia et al., 2010; Lara-Castillo et al., 2015). Increased nuclear *β*-catenin binds to the promoter region to promote Cx43 expression (Xia et al., 2010). In contrast, inhibition of PGE_2_ by a COX-2 inhibitor, Carprofen, blocks the activation of *β*-catenin nuclear translocation in osteocytes through the PI3K/Akt activation (Lara-Castillo et al., 2015). Canonical Wnt/β-catenin signaling is proven to stimulate anabolic actions in osteocytes (Osório, 2015; Tu et al., 2015). Deletion of *β*-catenin in osteocytes abolishes the bone anabolic response to mechanical loading (Javaheri et al., 2014; Kang et al., 2016). Thus, osteocytic accumulation of *β*-catenin may be one mechanism of mechanical-induced bone formation. In addition, the Wnt/β-catenin signaling is a well-known pathway associated with cell apoptosis (Ahmed et al., 1998). Mechanical stimulation-induced PGE_2_ in osteocytes blocks glucocorticoid-induced apoptosis through activated *β*-catenin, a downstream effector of the PI3K/Akt pathway (Kitase et al., 2010). In addition, the cAMP/PKA signaling pathway is involved in PGE_2_-mediated osteocyte survival during mechanical stimulation (Kitase et al., 2010). Integrins α5β1, which requires the opening of Cx43 hemichannels in osteocytes, participate in mechanical stimulation-induced osteocyte survival through FAK/Src and the ERK pathway (Plotkin et al., 2005).

The PGE_2_ released upon mechanical stimulation exerts a paracrine effect on the EP2/4 receptor to suppress the sclerostin expression through the cAMP/PKA pathway (Galea et al., 2011; Genetos et al., 2011). Sclerostin is an antagonist of canonical Wnt-β-catenin signaling in osteoblasts (Baron and Kneissel, 2013) through binding to the Wnt co-receptor Lrp5/6 (Li et al., 2005) to suppress osteogenesis (Sawakami et al., 2006). Several *in vitro* studies indicate the roles of osteocyte-derived PGE_2_ in promoting osteoblast activity during mechanical stimulation. Increased concentration of PGE_2_ released into the conditioned medium by fluid flow-loaded MLO-Y4 osteocytes promotes osteoblast differentiation (Zeng et al., 2019). In a 3D trabecular bone explant co-culture model, dynamic deformational loading can significantly increase the PGE_2_ release from osteocytes in their native extracellular matrix environment and promote osteoblastic bone formation (Chan et al., 2009). PGE_2_ released from osteocytes regulates osteoblast recruitment and collagen organization in the bone matrix during oscillatory fluid flow (Matsuzaka et al., 2021). Sclerostin not only inhibits bone formation but also promotes osteoclast formation. Osteocytes constitute a significant source of osteoclastogenic cytokine RANKL (Boyle et al., 2003; Xiong et al., 2011), which is also regulated by Wnt/β-catenin signaling (Donald et al., 2005; Nakashima et al., 2011). Sclerostin directly increases the levels of RANKL in osteocytes to regulate osteoclast activity by inhibiting *β*-catenin in osteoclasts (Wijenayaka et al., 2011). In contrast, sclerostin deficiency is resistant to bone resorption during mechanical unloading (Lin et al., 2009). Together, the PGE_2_ secreted from osteocytes upon mechanical stimulation has paracrine effects through EP2 and EP4 receptors to increase *β*-catenin and suppress sclerostin expression in osteocytes. Increased *β*-catenin in osteocytes promotes Cx43 expression, gap junction formation, mechanosensitivity, and survival of osteocytes. Suppression of sclerostin secretion promotes osteoblast activity and inhibits osteoblast activity.

### Cx43 hemichannels in bone development under physiological level of mechanical stress

Previous studies using Cx43 knockout and transgenic mouse models provide insightful information regarding the importance of Cx43 hemichannels in bone development under physiological mechanical conditions. Although global deleting Cx43 (Cx43^−/−^) caused early postnatal death due to an obstruction of the right ventricular outflow tract, they showed retarded intramembranous, endochondral ossification, craniofacial abnormalities, and osteoblast dysfunction (Lecanda et al., 2000; Thi et al., 2010; Chaible et al., 2011; Ishikawa et al., 2016). Moreover, mice with specific deletion of Cx43 by expressing the Cre recombinases in osteoprogenitors (*DM1*-Cre;*Cx43* ^-/flx^) (Watkins et al., 2011), preosteoblasts (*Osx1*-Cre;*Cx43* ^flx/flx^) (Hashida et al., 2014), early osteoblasts/osteocytes (*Col1α1*-Cre;*Cx43* ^-/flx^) (Castro et al., 2003; Chung et al., 2006), mature osteoblasts/osteocytes (*OCN*-Cre;*Cx43* ^-/flx^) (Bivi et al., 2012a), and osteocytes (8-kb *DMP1*-Cre;*Cx43* ^flx/flx^) (Bivi et al., 2012a; Bivi et al., 2012b) all showed thinner cortical thickness, larger marrow area, and total cross-sectional area. The change of cortical bone structure in these cKO mice was due to both increased periosteal osteoblastic bone formation (Watkins et al., 2011; Bivi et al., 2012a; Watkins et al., 2012; Pacheco-Costa et al., 2015) and an even greater unbalanced increase in endosteal osteoclastic bone resorption (Watkins et al., 2011; Bivi et al., 2012a; Watkins et al., 2012; Lloyd et al., 2013). In addition, Cx43 overexpressing in osteocytes (Cx43^OT^) preserves osteocyte viability and bone formation to ameliorate age-induced cortical bone loss (Davis et al., 2018). It is worth noting that Cx43 deficiency affects the production and release of PGE_2_ in osteoblasts and osteocytes. A lower amount of PGE_2_ is found in the primary calvaria cells of Cx43^−/−^ mice than in wild-type (WT) mice (Grimston et al., 2006). The absence of Cx43 in osteocytes by Cx43 shRNA attenuates PGE_2_ synthesis by COX-2 (Bivi et al., 2013). Although Cx43 knockout during bone developmental stages from the early stage (*DM1*-Cre) to the late stage (*DMP1*-Cre) of osteoblast differentiation shows the similar bone structure, Cx43 deficiency abolishes both Cx43 gap junctions and hemichannels. Thus, it is impossible to determine whether Cx43 gap junctions or/and hemichannels are responsible for the observed phenotypes. It is worth noting that other cell types may play indirect roles in osteoblast differentiation after knocking out Cx43. For example, Cx43 in osteoblasts/osteocytes indirectly modulates skeletal muscle growth and function (Shen et al., 2015; Li et al., 2021). In turn, skeletal muscle can also influence bone growth by releasing osteogenic myokines (Hamrick et al., 2010). Thus, the roles of Cx43 hemichannel in bone and other tissue crosstalk need to be further studied.

Besides Cx43-deficient mice, several Cx43 gene mutations can lead to a skeletal disease called oculodentodigital dysplasia (ODDD), with phenotypic presentations of syndactyly, craniofacial abnormalities, and long broad bones (Paznekas et al., 2003). To date, four mouse strains (Cx43^I130T/+^ (Kalcheva et al., 2007), Cx43^Jrt(G60S)/+^ (Flenniken et al., 2005), Cx43^G138R/+^ (Dobrowolski et al., 2008) and Cx43^K258Stop/-^ (Pacheco-Costa et al., 2015; Moorer et al., 2017)) with missense point mutations in one allele of the Cx43 gene are generated to mimic the phenotypes of ODDD. The mutations alter Cx43 protein conformation, thus leading to changed hemichannel activities (Dobrowolski et al., 2007). Increased (Dobrowolski et al., 2008) or decreased (Kalcheva et al., 2007) Cx43 hemichannel functions are found in these Cx43 mutants, indicating that normal Cx43 hemichannel function is crucial in maintaining bone structure.

Our group has generated two transgenic mouse models to dissect the function of Cx43 gap junctions and hemichannels in osteocytes, respectively. A *10 kb-DMP1* promoter drives the two transgenic mouse models R76W and ∆130–136 with the overexpression of dominant negative Cx43 mutants in osteocytes (Xu et al., 2015). The R76W site mutant, of which Cx43 amino acid residue arginine-76 (R) is replaced by tyrosine, inhibits gap junctions. The ∆130–136 mutant with the deletion of seven residues in the cytoplasmic loop of Cx43 protein at amino acids 130–136 inhibits both gap junctions and hemichannels. The bone phenotype in R76W mice is mostly like WT mice, except for increased endosteal osteoclast activity and bone remodeling markers in serum. In contrast, the ∆130–136 mice exhibit increased osteocyte apoptosis, endosteal resorption, and periosteal bone formation, resulting in higher tissue, cortical, and marrow cavity area of femoral midshaft at the femoral mid-diaphysis. The bone phenotypes in ∆130–136 mice are similar, but even more severe, than osteocyte-specific Cx43 cKO mice driven by 8-kb *DMP1* promoter (Bivi et al., 2012a), indicating Cx43 deficiency in osteocytes impairs the functions of Cx43 hemichannel in bone development. Compared to WT and R76W, the ∆130–136 mice show lower PGE_2_ levels in tibia diaphysis (Zhao et al., 2022a). As discussed above, PGE_2_ released by osteocytes mediated by Cx43 hemichannels is proven to maintain osteocyte survival and prevent their apoptosis (Kitase et al., 2010). Consistently, both ∆130–136 and 8-kb *DMP1*-Cre;*Cx43* cKO mice show increased osteocyte apoptosis in cortical bone. Impaired Cx43 hemichannels of osteocytes are also found in osteocyte-specific integrin a5 cKO mice driven by 10-kb *DMP1* promoter, which expresses lower serum PGE_2_ levels and increased osteocytes apoptosis and cortical thickness in tibias (Zhao et al., 2022b). Thus, under a physiological mechanical environment, Cx43 hemichannels and PGE_2_ in osteocytes likely play a predominant role in osteocyte vitality and bone structure.

### PGE_2_ release through osteocytic Cx43 hemichannels promotes bone anabolism upon mechanical loading

PGE_2_ stimulates bone resorption and formation (Jee and Ma, 1997; Blackwell et al., 2010). Continuous PGE_2_ treatment decreases cancellous bone mass due to bone resorption exceeding bone formation (Tian et al., 2007). Whereas moderate PGE_2_ treatment by intraperitoneal injection (Jee et al., 1985; Ueno et al., 1985) and local metaphyseal injection (Welch et al., 1993; Yang et al., 1993) increases both trabecular and cortical bone mass in growing rats. In addition, PGE_2_ prevents bone loss induced by ovariectomy (Mori et al., 1990; Harada et al., 1995), disuse (Jee et al., 1992), and orchidectomy (Li et al., 1995) in rats. Recent studies show that PGE_2_ activates EP4 in sensory nerves to promote bone formation and inhibit adipogenesis by inhibiting sympathetic activity through the central nervous system (Chen et al., 2019; Hu et al., 2020). The release of PGE_2_, a known direct product of bone mechanical stimulation, has important anabolic effects on the skeleton. In healthy women, a rapid and significant increase of PGE_2_ levels in the proximal tibial metaphysis is observed using the microdialysis technique in response to weight-bearing mechanical loading (Thorsen et al., 1996). Inhibition of PGE_2_ by a COX-2 inhibitor, NS-398, completely blocks tibial bone formation induced by four-point bending loading in rats (Forwood, 1996; Li et al., 2002). On the contrary, activation of the PGE_2_ receptor using ONO-4819 (agonist for prostaglandin E receptor subtype EP4) has an additive effect on bone formation in response to mechanical loading (Hagino et al., 2005). The involvement of Cx43 in the anabolic function of mechanical loading and PGE2 has been reported in earlier studies. Mice with Cx43 deficiency show altered bone anabolic response to mechanical loading. *Col1α1*-Cre;*Cx43* cKO mice show attenuated tibial endosteal bone formation during non-physiological four-point (Grimston et al., 2006) or three-point tibial bending (Grimston et al., 2008). A lower level of PGE_2_ is found in the primary calvaria cells from Cx43^−/−^ mice than in WT mice during mechanical stretching (Grimston et al., 2006). In *DM1*-Cre;*Cx43* cKO mice, axial tibia loading results in a greater decrease of endosteal bone formation compared to WT mice (Grimston et al., 2012). However, Cx43 deficiency has a positive effect on periosteal bone formation. Deletion of Cx43 in osteoblasts/osteocytes *(DM1*-Cre;*Cx43* cKO and *OCN*-Cre;*Cx43* cKO mice) showed an enhanced tibial periosteal response to tibial axial compression (Grimston et al., 2012) or tibial cantilever bending (Zhang et al., 2011). Similarly, deletion of Cx43 in osteocytes (*DMP1*-*Cre*; *Cx43* cKO mice) showed enhanced periosteal bone formation in response to ulna compression (Bivi et al., 2013). Nevertheless, these cKO mice have both impaired gap junctions and hemichannels, as well as potential channel-independent roles of Cx43. Thus, the specific role of gap junctions and hemichannels formed by Cx43 hemichannels in response to mechanical loading in the bone cannot be resolved with Cx43 deletion models.

Recently, our group find a close relationship between Cx43 hemichannels and PGE_2_ release in skeletal response to mechanical loading *in vivo*. In this study, PGE_2_ levels in the diaphysis are significantly increased in WT and gap junction impaired R76W upon tibial cyclic compression loading. Increased PGE_2_ level suppresses the sclerostin expression in osteocytes and bone formation on the endosteal surface. However, Δ130-136 mice with impaired gap junctions and hemichannels show unchanged PGE_2_ levels and sclerostin expression in osteocytes. As a result, the increased bone mass caused by mechanical loading is not seen in Δ130-136 mice. Attenuated bone formation and increased resorption on the endosteal surface lead to the enlargement of the bone marrow cavity and inhibited bone mass gain (Zhao et al., 2022a). The data points to the role of Cx43 hemichannels in mediating PGE_2_ release and the anabolic action of mechanical loading. To further investigate whether the changed mechanical response in Δ130-136 mice is due to Cx43 hemichannels, a specific mouse monoclonal blocking antibody Cx43 (M1) was developed and used to investigate the role of Cx43 hemichannels *in vivo.* Administration of this particular antibody exhibits similar effects as Δ130-136 mice, including the attenuated PGE_2_ level and inhibited anabolic response to mechanical loading on the endosteal surface. PGE_2_ administration, however, can rescue the attenuated endosteal osteogenic response to mechanical loading impeded by the Cx43 (M1) antibody. PGE_2_ acts in a paracrine manner to suppress sclerostin expression *in vitro* (Galea et al., 2011; Genetos et al., 2011). These *in vivo* studies demonstrate that Cx43 hemichannels activated by mechanical stimulation release PGE_2_ from osteocytes to suppress sclerostin expression in osteocytes and enhance osteoblast activity and bone formation on endosteal surfaces. We further demonstrate the important role of Cx43 hemichannels and PGE_2_ release in bone anabolic response to mechanical loading through the use of osteocyte-specific integrin a5 cKO mice driven by a 10-kb *DMP1* promoter (Zhao et al., 2022b). Since the interaction between integrin α5 and Cx43 is essential for the hemichannel opening by mechanical loading (Batra et al., 2012), integrin α5 deficiency impedes load-induced Cx43 hemichannel opening and PGE_2_ release (Batra et al., 2012; Zhao et al., 2022b). Integrin α5 cKO mice in osteocytes exhibit attenuated loading effects on catabolic sclerostin reduction and anabolic *β*-catenin increase, contributing to decreased endosteal osteoblasts and bone formation (Figure 1). Our studies show that the anabolic effect of Cx43 hemichannel-released PGE_2_ is on the endosteal surface (Zhao et al., 2022a; Zhao et al., 2022b). Consistently, previous studies also report that the effect of PGE_2_ on bone anabolic response to mechanical loading appears to be more on the endosteal surface than the periosteal surface (Forwood, 1996; Li et al., 2002; Hagino et al., 2005).

Interestingly, inhibited Cx43 hemichannels in Δ130-136 mice, Cx43 (M1) group, and integrin α5 cKO mice showed an increase of osteoclast activity on the endosteal surface during mechanical loading. Whether this catabolic response is related to PGE_2_ requires further investigation. The mechanosensitivity of bone is gradually diminished during aging (Holguin et al., 2014; Holguin et al., 2016), accompanied by impaired mechanotransduction (Chalil et al., 2015) and decreased Cx43 expression in osteocytes (Kar et al., 2013). We speculate that attenuated Cx43 expression in osteocytes is related to abolished Cx43 hemichannel activity and PGE_2_ release in aged bone. Indeed, significantly reduced PGE_2_ EP4 receptors in the sensory nerve of aged bone attenuate the sensibility to changes in bone metabolism (Lv et al., 2022).

### Cx43 hemichannels play a protective role against bone loss during mechanical unloading

Reduced or no mechanical loading, such as long bed rest (Spector et al., 2009), and astronauts in space missions (Keyak et al., 2009), harms skeletal health, which is associated with an imbalanced bone turnover. Suppressed osteoblastic bone formation and activated osteoclastic bone resorption (Bikle and Halloran, 1999; Kondo et al., 2005) increase bone loss and fracture risk. Previous *in vitro* studies show that Cx43 hemichannels participate in response to mechanical unloading. Zero-gravity by parabolic flight decreases Cx43 expression in osteocytes (Di et al., 2011). Simulated microgravity by a random position machine (RPM) increases the activity of Cx43 hemichannels and the release of PGE_2_ (Xu et al., 2017). Dominant negative integrin β1 mutants driven by an *OCN* promoter show a lower cancellous bone mass in the distal femoral metaphysis caused by increased bone resorption and decreased bone formation during short-term hindlimb unloading, a model mimicking unloading/disuse (Iwaniec et al., 2005). Given that integrin a5β1 regulates the opening of the Cx43 hemichannels, this study implies the vital role of Cx43 hemichannels in bone response to unloading. High extracellular PGE_2_ due to sustained opening of hemichannels during unloading (Xu et al., 2017) resulted in osteoclast resorption and bone loss (Coon et al., 2007; Knippenberg et al., 2007; Tian et al., 2007). Consistently, deletion of Cx43 in osteoblast/osteocyte-specific Cx43 cKO mice driven by the 2.3-kb *Col1α1* promoter (Grimston et al., 2011) and *OCN* promoter (Lloyd et al., 2012; Lloyd et al., 2013) show protection against unloading-induced bone loss. In contrast, our previous study shows that enhanced Cx43 hemichannels in R76W mice protect from osteocyte apoptosis in cortical bone during mechanical unloading (Zhao et al., 2020). PGE_2_ is an inhibitor of sclerostin expression, and both sclerostin and PGE_2_ inhibitors are known to be associated with cell apoptosis (Ahmed et al., 1998). It is assumed that PGE_2_ from Cx43 hemichannels has a protective role against osteocyte apoptosis. Multifaced roles of Cx43 could cause the difference seen between Cx43 deletion and hemichannel-impaired models. Further studies are needed to establish the underlying mechanisms of Cx43 hemichannels and PGE_2_ in response to mechanical unloading.

## Future perspectives

With research advances and the development of new transgenic mouse models, the inter-relationship between Cx43 hemichannels and extracellular PGE_2_ in mediating osteogenic response to mechanical loading is becoming more evident. However, several questions remain, including 1) if extracellular PGE_2_ released by hemichannels upon mechanical loading has any direct action on osteoclasts; 2) if the loading-induced bone anabolic response is partly regulated through the influence of osteocyte-released PGE_2_ on the nerve system. Early studies reported that temporarily blocking peripheral neurons by bupivacaine reduces bone formation in compressed ulna (Sample et al., 2008). Recently, PGE_2_ has been found to act on sensory neurons and affect sympathetic nerve activity, and regulate bone homeostasis (Chen et al., 2019; Lv et al., 2021). It is speculated that the PGE_2_ released from Cx43 hemichannels in osteocytes may not only have autocrine and paracrine effects on osteocytes and the other types of bone cells, respectively, but may also systematically influence sensory nerves. The interactions of bone cells and sensory neurons regulated by PGE_2_ remain largely unknown. These could all be potential directions for future research. Moreover, further research on unveiling the mechanism of action for hemichannels and PGE_2_ may help in the discovery and development of potential therapeutics that aid in treating bone loss, in particular, in the elderly population with lost sensitivity to anabolic responses to mechanical stimulation.

## References

[B1] Aberle H. Bauer A. Stappert J. Kispert A. Kemler R. (1997). beta-catenin is a target for the ubiquitin-proteasome pathway. Embo J. 16, 3797–3804. 10.1093/emboj/16.13.3797 9233789 PMC1170003

[B2] Ahmed Y. Hayashi S. Levine A. Wieschaus E. (1998). Regulation of armadillo by a Drosophila APC inhibits neuronal apoptosis during retinal development. Cell. 93, 1171–1182. 10.1016/s0092-8674(00)81461-0 9657150

[B3] Ajubi N. E. Klein-Nulend J. Alblas M. J. Burger E. H. Nijweide P. J. (1999). Signal transduction pathways involved in fluid flow-induced PGE2 production by cultured osteocytes. Am. J. Physiol. 276, e171–e178. 10.1152/ajpendo.1999.276.1.E171 9886964

[B4] Ajubi N. E. Klein-Nulend J. Nijweide P. J. Vrijheid-Lammers T. Alblas M. J. Burger E. H. (1996). Pulsating fluid flow increases prostaglandin production by cultured chicken osteocytes--a cytoskeleton-dependent process. Biochem. Biophys. Res. Commun. 225, 62–68. 10.1006/bbrc.1996.1131 8769095

[B6] Bakker A. D. Klein-Nulend J. Burger E. H. (2003). Mechanotransduction in bone cells proceeds via activation of COX-2, but not COX-1. Biochem. Biophys. Res. Commun. 305, 677–683. 10.1016/s0006-291x(03)00831-3 12763047

[B7] Baron R. Kneissel M. (2013). WNT signaling in bone homeostasis and disease: From human mutations to treatments. Nat. Med. 19, 179–192. 10.1038/nm.3074 23389618

[B8] Batra N. Burra S. Siller-Jackson A. J. Gu S. Xia X. Weber G. F. (2012). Mechanical stress-activated integrin α5β1 induces opening of connexin 43 hemichannels. Proc. Natl. Acad. Sci. U. S. A. 109, 3359–3364. 10.1073/pnas.1115967109 22331870 PMC3295295

[B9] Batra N. Riquelme M. A. Burra S. Jiang J. X. (2014a). 14-3-3θ facilitates plasma membrane delivery and function of mechanosensitive connexin 43 hemichannels. J. Cell. Sci. 127, 137–146. 10.1242/jcs.133553 24163432 PMC3874784

[B10] Batra N. Riquelme M. A. Burra S. Kar R. Gu S. Jiang J. X. (2014b). Direct regulation of osteocytic connexin 43 hemichannels through AKT kinase activated by mechanical stimulation. J. Biol. Chem. 289, 10582–10591. 10.1074/jbc.M114.550608 24563481 PMC4036178

[B11] Bikle D. D. Halloran B. P. (1999). The response of bone to unloading. J. Bone Min. Metab. 17, 233–244. 10.1007/s007740050090 10575587

[B12] Bivi N. Condon K. W. Allen M. R. Farlow N. Passeri G. Brun L. R. (2012a). Cell autonomous requirement of connexin 43 for osteocyte survival consequences for endocortical resorption and periosteal bone formation. J. Bone Min. Res. 27, 374–389. 10.1002/jbmr.548 PMC3271138 22028311

[B13] Bivi N. Nelson M. T. Faillace M. E. Li J. Miller L. M. Plotkin L. I. (2012b). Deletion of Cx43 from osteocytes results in defective bone material properties but does not decrease extrinsic strength in cortical bone. Calcif. Tissue Int. 91, 215–224. 10.1007/s00223-012-9628-z 22865265 PMC3729333

[B14] Bivi N. Pacheco-Costa R. Brun L. R. Murphy T. R. Farlow N. R. Robling A. G. (2013). Absence of Cx43 selectively from osteocytes enhances responsiveness to mechanical force in mice. J. Orthop. Res. 31, 1075–1081. 10.1002/jor.22341 23483620 PMC3663897

[B15] Blackwell K. A. Raisz L. G. Pilbeam C. C. (2010). Prostaglandins in bone: Bad cop, good cop? Trends Endocrinol. Metab. 21, 294–301. 10.1016/j.tem.2009.12.004 20079660 PMC2862787

[B17] Bonewald L. F. (2011). The amazing osteocyte. J. Bone Min. Res. 26, 229–238. 10.1002/jbmr.320 PMC3179345 21254230

[B18] Boyle W. J. Simonet W. S. Lacey D. L. (2003). Osteoclast differentiation and activation. Nature 423, 337–342. 10.1038/nature01658 12748652

[B19] Burra S. Nicolella D. P. Francis W. L. Freitas C. J. Mueschke N. J. Poole K. (2010). Dendritic processes of osteocytes are mechanotransducers that induce the opening of hemichannels. Proc. Natl. Acad. Sci. U. S. A. 107, 13648–13653. 10.1073/pnas.1009382107 20643964 PMC2922284

[B20] Castro C. H. Stains J. P. Sheikh S. Szejnfeld V. L. Willecke K. Theis M. (2003). Development of mice with osteoblast-specific connexin43 gene deletion. Cell. Commun. Adhes. 10, 445–450. 10.1080/cac.10.4-6.445.450 14681055

[B21] Chaible L. M. Sanches D. S. Cogliati B. Mennecier G. Dagli M. L. (2011). Delayed osteoblastic differentiation and bone development in Cx43 knockout mice. Toxicol. Pathol. 39, 1046–1055. 10.1177/0192623311422075 21934140

[B22] Chalil S. Jaspers R. T. Manders R. J. Klein-Nulend J. Bakker A. D. Deldicque L. (2015). Increased endoplasmic reticulum stress in mouse osteocytes with aging alters Cox-2 response to mechanical stimuli. Calcif. Tissue Int. 96, 123–128. 10.1007/s00223-014-9944-6 25539857

[B23] Chan M. E. Lu X. L. Huo B. Baik A. D. Chiang V. Guldberg R. E. (2009). A trabecular bone explant model of osteocyte-osteoblast Co-culture for bone mechanobiology. Cell. Mol. Bioeng. 2, 405–415. 10.1007/s12195-009-0075-5 20827376 PMC2935082

[B24] Chen H. Hu B. Lv X. Zhu S. Zhen G. Wan M. (2019). Prostaglandin E2 mediates sensory nerve regulation of bone homeostasis. Nat. Commun. 10, 181. 10.1038/s41467-018-08097-7 30643142 PMC6331599

[B25] Cheng B. Kato Y. Zhao S. Luo J. Sprague E. Bonewald L. F. (2001b). PGE(2) is essential for gap junction-mediated intercellular communication between osteocyte-like MLO-Y4 cells in response to mechanical strain. Endocrinology 142, 3464–3473. 10.1210/endo.142.8.8338 11459792

[B26] Cheng B. Zhao S. Luo J. Sprague E. Bonewald L. F. Jiang J. X. (2001a). Expression of functional gap junctions and regulation by fluid flow in osteocyte-like MLO-Y4 cells. J. Bone Min. Res. 16, 249–259. 10.1359/jbmr.2001.16.2.249 11204425

[B27] Cherian P. P. Cheng B. Gu S. Sprague E. Bonewald L. F. Jiang J. X. (2003). Effects of mechanical strain on the function of Gap junctions in osteocytes are mediated through the prostaglandin EP2 receptor. J. Biol. Chem. 278, 43146–43156. 10.1074/jbc.M302993200 12939279

[B28] Cherian P. P. Siller-Jackson A. J. Gu S. Wang X. Bonewald L. F. Sprague E. (2005). Mechanical strain opens connexin 43 hemichannels in osteocytes: A novel mechanism for the release of prostaglandin. Mol. Biol. Cell. 16, 3100–3106. 10.1091/mbc.e04-10-0912 15843434 PMC1165395

[B29] Chung D. J. Castro C. H. Watkins M. Stains J. P. Chung M. Y. Szejnfeld V. L. (2006). Low peak bone mass and attenuated anabolic response to parathyroid hormone in mice with an osteoblast-specific deletion of connexin43. J. Cell. Sci. 119, 4187–4198. 10.1242/jcs.03162 16984976

[B30] Civitelli R. Beyer E. C. Warlow P. M. Robertson A. J. Geist S. T. Steinberg T. H. (1993). Connexin43 mediates direct intercellular communication in human osteoblastic cell networks. J. Clin. Invest. 91, 1888–1896. 10.1172/JCI116406 8387535 PMC288182

[B31] Civitelli R. (2008). Cell-cell communication in the osteoblast/osteocyte lineage. Arch. Biochem. Biophys. 473, 188–192. 10.1016/j.abb.2008.04.005 18424255 PMC2441851

[B32] Coon D. Gulati A. Cowan C. He J. (2007). The role of cyclooxygenase-2 (COX-2) in inflammatory bone resorption. J. Endod. 33, 432–436. 10.1016/j.joen.2006.12.001 17368333

[B33] Davis H. M. Aref M. W. Aguilar-Perez A. Pacheco-Costa R. Allen K. Valdez S. (2018). Cx43 overexpression in osteocytes prevents osteocyte apoptosis and preserves cortical bone quality in aging mice. JBMR Plus 2, 206–216. 10.1002/jbm4.10035 29978155 PMC6029870

[B34] Di S. M. Qian A. R. Qu L. N. Zhang W. Wang Z. Ding C. (2011). Graviresponses of osteocytes under altered gravity. Adv. Space Res. 48, 1161–1166. 10.1016/j.asr.2011.05.030

[B35] Dobrowolski R. Sasse P. Schrickel J. W. Watkins M. Kim J. S. Rackauskas M. (2008). The conditional connexin43G138R mouse mutant represents a new model of hereditary oculodentodigital dysplasia in humans. Hum. Mol. Genet. 17, 539–554. 10.1093/hmg/ddm329 18003637 PMC2847779

[B36] Dobrowolski R. Sommershof A. Willecke K. (2007). Some oculodentodigital dysplasia-associated Cx43 mutations cause increased hemichannel activity in addition to deficient gap junction channels. J. Membr. Biol. 219, 9–17. 10.1007/s00232-007-9055-7 17687502

[B37] Donald P. B. Glass A. II Ahn J. D. Starbuck M. Patel M. S. Clevers H. (2005). Canonical Wnt signaling in differentiated osteoblasts controls osteoclast differentiation. Dev. Cell. 8, 751–764. 10.1016/j.devcel.2005.02.017 15866165

[B38] Dong L. Song Y. Zhang Y. Zhao W. Wang C. Lin H. (2021). Mechanical stretch induces osteogenesis through the alternative activation of macrophages. J. Cell. Physiol. 236, 6376–6390. 10.1002/jcp.30312 33634492

[B39] Duncan R. L. Turner C. H. (1995). Mechanotransduction and the functional response of bone to mechanical strain. Calcif. Tissue Int. 57, 344–358. 10.1007/bf00302070 8564797

[B40] Erlandson M. C. Kontulainen S. A. Chilibeck P. D. Arnold C. M. Faulkner R. A. Baxter-Jones A. D. (2012). Higher premenarcheal bone mass in elite gymnasts is maintained into young adulthood after long-term retirement from sport: A 14-year follow-up. J. Bone Min. Res. 27, 104–110. 10.1002/jbmr.514 21956460

[B41] Flenniken A. M. Osborne L. R. Anderson N. Ciliberti N. Fleming C. Gittens J. E. (2005). A Gja1 missense mutation in a mouse model of oculodentodigital dysplasia. Development 132, 4375–4386. 10.1242/dev.02011 16155213

[B42] Forwood M. R. (1996). Inducible cyclo-oxygenase (COX-2) mediates the induction of bone formation by mechanical loading *in vivo* . J. Bone Min. Res. 11, 1688–1693. 10.1002/jbmr.5650111112 8915776

[B43] Furuyashiki T. Narumiya S. (2011). Stress responses: The contribution of prostaglandin E(2) and its receptors. Nat. Rev. Endocrinol. 7, 163–175. 10.1038/nrendo.2010.194 21116297

[B44] Galea G. L. Sunters A. Meakin L. B. Zaman G. Sugiyama T. Lanyon L. E. (2011). Sost down-regulation by mechanical strain in human osteoblastic cells involves PGE2 signaling via EP4. FEBS Lett. 585, 2450–2454. 10.1016/j.febslet.2011.06.019 21723865 PMC3149668

[B45] Genetos D. C. Geist D. J. Liu D. Donahue H. J. Duncan R. L. (2005). Fluid shear-induced ATP secretion mediates prostaglandin release in MC3T3-E1 osteoblasts. J. Bone Min. Res. 20, 41–49. 10.1359/JBMR.041009 PMC2929123 15619668

[B46] Genetos D. C. Kephart C. J. Zhang Y. Yellowley C. E. Donahue H. J. (2007). Oscillating fluid flow activation of gap junction hemichannels induces ATP release from MLO-Y4 osteocytes. J. Cell. Physiol. 212, 207–214. 10.1002/jcp.21021 17301958 PMC2929812

[B47] Genetos D. C. Yellowley C. E. Loots G. G. (2011). Prostaglandin E2 signals through PTGER2 to regulate sclerostin expression. PLoS One 6, e17772. 10.1371/journal.pone.0017772 21436889 PMC3059227

[B48] Goodenough D. A. Goliger J. A. Paul D. L. (1996). Connexins, connexons, and intercellular communication. Annu. Rev. Biochem. 65, 475–502. 10.1146/annurev.bi.65.070196.002355 8811187

[B49] Grimston S. K. Brodt M. D. Silva M. J. Civitelli R. (2008). Attenuated response to *in vivo* mechanical loading in mice with conditional osteoblast ablation of the connexin43 gene (Gja1). J. Bone Min. Res. 23, 879–886. 10.1359/jbmr.080222 PMC2677086 18282131

[B50] Grimston S. K. Goldberg D. B. Watkins M. Brodt M. D. Silva M. J. Civitelli R. (2011). Connexin43 deficiency reduces the sensitivity of cortical bone to the effects of muscle paralysis. J. Bone Min. Res. 26, 2151–2160. 10.1002/jbmr.425 PMC3306012 21590735

[B51] Grimston S. K. Screen J. Haskell J. H. Chung D. J. Brodt M. D. Silva M. J. (2006). Role of connexin43 in osteoblast response to physical load. Ann. N. Y. Acad. Sci. 1068, 214–224. 10.1196/annals.1346.023 16831921

[B52] Grimston S. K. Watkins M. P. Brodt M. D. Silva M. J. Civitelli R. (2012). Enhanced periosteal and endocortical responses to axial tibial compression loading in conditional connexin43 deficient mice. PLoS One 7, e44222. 10.1371/journal.pone.0044222 22970183 PMC3438198

[B53] Hagino H. Kuraoka M. Kameyama Y. Okano T. Teshima R. (2005). Effect of a selective agonist for prostaglandin E receptor subtype EP4 (ONO-4819) on the cortical bone response to mechanical loading. Bone 36, 444–453. 10.1016/j.bone.2004.12.013 15777678

[B150] Hamrick M. W. McNeil P. L. Patterson S. L. (2010). Role of muscle-derived growth factors in bone formation. J. Musculoskelet. Neuronal Interact. 10, 64–70. 20190381 PMC3753580

[B54] Harada S. I. Balena R. Rodan G. A. Rodan S. B. (1995). The role of prostaglandins in bone formation. Connect. Tissue Res. 31, 279–282. 10.3109/03008209509010823 15612368

[B55] Hashida Y. Nakahama K. Shimizu K. Akiyama M. Harada K. Morita I. (2014). Communication-dependent mineralization of osteoblasts via gap junctions. Bone 61, 19–26. 10.1016/j.bone.2013.12.031 24389413

[B56] Holguin N. Brodt M. D. Sanchez M. E. Silva M. J. (2014). Aging diminishes lamellar and woven bone formation induced by tibial compression in adult C57BL/6. Bone 65, 83–91. 10.1016/j.bone.2014.05.006 24836737 PMC4091978

[B57] Holguin N. Brodt M. D. Silva M. J. (2016). Activation of Wnt signaling by mechanical loading is impaired in the bone of old mice. J. Bone Min. Res. 31, 2215–2226. 10.1002/jbmr.2900 PMC5397287 27357062

[B58] Hu B. Lv X. Chen H. Xue P. Gao B. Wang X. (2020). Sensory nerves regulate mesenchymal stromal cell lineage commitment by tuning sympathetic tones. J. Clin. Invest. 130, 3483–3498. 10.1172/JCI131554 32191640 PMC7324175

[B59] Ilvesaro J. Tuukkanen J. (2003). Gap-junctional regulation of osteoclast function. Crit. Rev. Eukaryot. Gene Expr. 13, 133–146. 10.1615/critreveukaryotgeneexpr.v13.i24.70 14696962

[B60] Ilvesaro J. Väänänen K. Tuukkanen J. (2000). Bone-resorbing osteoclasts contain gap-junctional connexin-43. J. Bone Min. Res. 15, 919–926. 10.1359/jbmr.2000.15.5.919 10804022

[B61] Imamura K. Ozawa H. Hiraide T. Takahashi N. Shibasaki Y. Fukuhara T. (1990). Continuously applied compressive pressure induces bone resorption by a mechanism involving prostaglandin E2 synthesis. J. Cell. Physiol. 144, 222–228. 10.1002/jcp.1041440207 2166056

[B62] Ishikawa M. Williams G. L. Ikeuchi T. Sakai K. Fukumoto S. Yamada Y. (2016). Pannexin 3 and connexin 43 modulate skeletal development through their distinct functions and expression patterns. J. Cell. Sci. 129, 1018–1030. 10.1242/jcs.176883 26759176 PMC4813316

[B63] Iwaniec U. T. Wronski T. J. Amblard D. Nishimura Y. van der Meulen M. C. Wade C. E. (2005). Effects of disrupted β_1_-integrin function on the skeletal response to short-term hindlimb unloading in mice. J. Appl. Physiol. 98, 690–696. 10.1152/japplphysiol.00689.2004 15465888

[B64] Javaheri B. Stern A. R. Lara N. Dallas M. Zhao H. Liu Y. (2014). Deletion of a single β-catenin allele in osteocytes abolishes the bone anabolic response to loading. J. Bone Min. Res. 29, 705–715. 10.1002/jbmr.2064 PMC4171742 23929793

[B65] Jee W. S. Akamine T. Ke H. Z. Li X. J. Tang L. Y. Zeng Q. Q. (1992). Prostaglandin E2 prevents disuse-induced cortical bone loss. Bone 13, 153–159. 10.1016/8756-3282(92)90005-h 1576011

[B66] Jee W. S. Ma Y. F. (1997). The *in vivo* anabolic actions of prostaglandins in bone. Bone 21, 297–304. 10.1016/s8756-3282(97)00147-6 9315332

[B67] Jee W. S. Ueno K. Deng Y. P. Woodbury D. M. (1985). The effects of prostaglandin E2 in growing rats: Increased metaphyseal hard tissue and cortico-endosteal bone formation. Calcif. Tissue Int. 37, 148–157. 10.1007/BF02554834 3924371

[B68] Jiang J. X. Cheng B. (2001). Mechanical stimulation of gap junctions in bone osteocytes is mediated by prostaglandin E2. Cell. Commun. Adhes. 8, 283–288. 10.3109/15419060109080738 12064603

[B69] Jiang J. X. Cherian P. P. (2003). Hemichannels formed by connexin 43 play an important role in the release of prostaglandin E(2) by osteocytes in response to mechanical strain. Cell. Commun. Adhes. 10, 259–264. 10.1080/cac.10.4-6.259.264 14681026

[B70] Jiang W. Jin Y. Zhang S. Ding Y. Huo K. Yang J. (2022). PGE2 activates EP4 in subchondral bone osteoclasts to regulate osteoarthritis. Bone Res. 10, 27. 10.1038/s41413-022-00201-4 35260562 PMC8904489

[B71] Johnson D. L. McAllister T. N. Frangos J. A. (1996). Fluid flow stimulates rapid and continuous release of nitric oxide in osteoblasts. Am. J. Physiol. 271, e205–e208. 10.1152/ajpendo.1996.271.1.E205 8760099

[B72] Joldersma M. Burger E. H. Semeins C. M. Klein-Nulend J. (2000). Mechanical stress induces COX-2 mRNA expression in bone cells from elderly women. J. Biomech. 33, 53–61. 10.1016/s0021-9290(99)00172-4 10609518

[B73] Kalcheva N. Qu J. Sandeep N. Garcia L. Zhang J. Wang Z. (2007). Gap junction remodeling and cardiac arrhythmogenesis in a murine model of oculodentodigital dysplasia. Proc. Natl. Acad. Sci. U. S. A. 104, 20512–20516. 10.1073/pnas.0705472105 18077386 PMC2154462

[B74] Kamel M. A. Picconi J. L. Lara-Castillo N. Johnson M. L. (2010). Activation of β-catenin signaling in MLO-Y4 osteocytic cells versus 2T3 osteoblastic cells by fluid flow shear stress and PGE2: Implications for the study of mechanosensation in bone. Bone 47, 872–881. 10.1016/j.bone.2010.08.007 20713195 PMC2952691

[B75] Kang K. S. Hong J. M. Robling A. G. (2016). Postnatal β-catenin deletion from Dmp1-expressing osteocytes/osteoblasts reduces structural adaptation to loading, but not periosteal load-induced bone formation. Bone 88, 138–145. 10.1016/j.bone.2016.04.028 27143110 PMC4899196

[B76] Kar R. Riquelme M. A. Werner S. Jiang J. X. (2013). Connexin 43 channels protect osteocytes against oxidative stress-induced cell death. J. Bone Min. Res. 28, 1611–1621. 10.1002/jbmr.1917 PMC3688648 23456878

[B77] Keyak J. H. Koyama A. K. LeBlanc A. Lu Y. Lang T. F. (2009). Reduction in proximal femoral strength due to long-duration spaceflight. Bone 44, 449–453. 10.1016/j.bone.2008.11.014 19100348

[B78] Kitase Y. Barragan L. Qing H. Kondoh S. Jiang J. X. Johnson M. L. (2010). Mechanical induction of PGE_2_ in osteocytes blocks glucocorticoid induced apoptosis through both the β-catenin and PKA pathways. J. Bone Min. Res. 25, 2657–2668. 10.1002/jbmr.168 PMC3179278 20578217

[B79] Klein-Nulend J. Burger E. H. Semeins C. M. Raisz L. G. Pilbeam C. C. (1997). Pulsating fluid flow stimulates prostaglandin release and inducible prostaglandin G/H synthase mRNA expression in primary mouse bone cells. J. Bone Min. Res. 12, 45–51. 10.1359/jbmr.1997.12.1.45 9240724

[B80] Knippenberg M. Helder M. N. de Blieck-Hogervorst J. M. Wuisman P. I. Klein-Nulend J. (2007). Prostaglandins differentially affect osteogenic differentiation of human adipose tissue-derived mesenchymal stem cells. Tissue Eng. 13, 2495–2503. 10.1089/ten.2006.0420 17655490

[B81] Kondo H. Nifuji A. Takeda S. Ezura Y. Rittling S. R. Denhardt D. T. (2005). Unloading induces osteoblastic cell suppression and osteoclastic cell activation to lead to bone loss via sympathetic nervous system. J. Biol. Chem. 280, 30192–30200. 10.1074/jbc.M504179200 15961387

[B82] Kumar N. M. Gilula N. B. (1996). The gap junction communication channel. Cell. 84, 381–388. 10.1016/s0092-8674(00)81282-9 8608591

[B83] Lang T. LeBlanc A. Evans H. Lu Y. Genant H. Yu A. (2004). Cortical and trabecular bone mineral loss from the spine and hip in long-duration spaceflight. J. Bone Min. Res. 19, 1006–1012. 10.1359/JBMR.040307 15125798

[B84] Lara-Castillo N. Kim-Weroha N. A. Kamel M. A. Javaheri B. Ellies D. L. Krumlauf R. E. (2015). *In vivo* mechanical loading rapidly activates β-catenin signaling in osteocytes through a prostaglandin mediated mechanism. Bone 76, 58–66. 10.1016/j.bone.2015.03.019 25836764 PMC4447591

[B85] Lecanda F. Warlow P. M. Sheikh S. Furlan F. Steinberg T. H. Civitelli R. (2000). Connexin43 deficiency causes delayed ossification, craniofacial abnormalities, and osteoblast dysfunction. J. Cell. Biol. 151, 931–944. 10.1083/jcb.151.4.931 11076975 PMC2169447

[B148] Li X. Han L. Nookaew I. Mannen E. Silva M. J. Almeida M. (2019). Stimulation of Piezo1 by mechanical signals promotes bone anabolism. eLife 8, e49631. 10.7554/eLife.49631 31588901 PMC6779475

[B86] Li G. Zhang L. Ning K. Yang B. Acosta F. M. Shang P. (2021). Osteocytic Connexin43 channels regulate bone-muscle crosstalk. Cells 10, 237. 10.3390/cells10020237 33530465 PMC7911162

[B87] Li J. Burr D. B. Turner C. H. (2002). Suppression of prostaglandin synthesis with NS-398 has different effects on endocortical and periosteal bone formation induced by mechanical loading. Calcif. Tissue Int. 70, 320–329. 10.1007/s00223-001-1025-y 12004337

[B88] Li M. Jee W. S. Ke H. Z. Tang L. Y. Ma Y. F. Liang X. G. (1995). Prostaglandin E2 administration prevents bone loss induced by orchidectomy in rats. J. Bone Min. Res. 10, 66–73. 10.1002/jbmr.5650100111 7747632

[B89] Li X. Zhang Y. Kang H. Liu W. Liu P. Zhang J. (2005). Sclerostin binds to LRP5/6 and antagonizes canonical Wnt signaling. J. Biol. Chem. 280, 19883–19887. 10.1074/jbc.M413274200 15778503

[B90] Lin C. Jiang X. Dai Z. Guo X. Weng T. Wang J. (2009). Sclerostin mediates bone response to mechanical unloading through antagonizing Wnt/beta-catenin signaling. J. Bone Min. Res. 24, 1651–1661. 10.1359/jbmr.090411 19419300

[B91] Liu P. Tu J. Wang W. Li Z. Li Y. Yu X. (2022). Effects of mechanical stress stimulation on function and expression mechanism of osteoblasts. Front. Bioeng. Biotechnol. 10, 830722. 10.3389/fbioe.2022.830722 35252138 PMC8893233

[B92] Lloyd S. A. Lewis G. S. Zhang Y. Paul E. M. Donahue H. J. (2012). Connexin 43 deficiency attenuates loss of trabecular bone and prevents suppression of cortical bone formation during unloading. J. Bone Min. Res. 27, 2359–2372. 10.1002/jbmr.1687 PMC3683470 22714552

[B93] Lloyd S. A. Loiselle A. E. Zhang Y. Donahue H. J. (2013). Connexin 43 deficiency desensitizes bone to the effects of mechanical unloading through modulation of both arms of bone remodeling. Bone 57, 76–83. 10.1016/j.bone.2013.07.022 23891909 PMC4480865

[B94] Lv X. Gao F. Cao X. (2022). Skeletal interoception in bone homeostasis and pain. Cell. Metab. 34 (12), 1914–1931. 10.1016/j.cmet.2022.09.025 36257317 PMC9742337

[B95] Lv X. Gao F. Li T. P. Xue P. Wang X. Wan M. (2021). Skeleton interoception regulates bone and fat metabolism through hypothalamic neuroendocrine NPY. eLife 10, e70324. 10.7554/eLife.70324 34468315 PMC8439655

[B96] Matsuzaka T. Matsugaki A. Nakano T. (2021). Control of osteoblast arrangement by osteocyte mechanoresponse through prostaglandin E2 signaling under oscillatory fluid flow stimuli. Biomaterials 279, 121203. 10.1016/j.biomaterials.2021.121203 34717197

[B97] Miwa M. Kozawa O. Tokuda H. Kawakubo A. Yoneda M. Oiso Y. (1991). Effects of hypergravity on proliferation and differentiation of osteoblast-like cells. Bone Min. 14, 15–25. 10.1016/0169-6009(91)90099-l 1651138

[B98] Moorer M. C. Hebert C. Tomlinson R. E. Iyer S. R. Chason M. Stains J. P. (2017). Defective signaling, osteoblastogenesis and bone remodeling in a mouse model of connexin 43 C-terminal truncation. J. Cell. Sci. 130, 531–540. 10.1242/jcs.197285 28049723 PMC5312734

[B99] Mori S. Jee W. S. Li X. J. Chan S. Kimmel D. B. (1990). Effects of prostaglandin E2 on production of new cancellous bone in the axial skeleton of ovariectomized rats. Bone 11, 103–113. 10.1016/8756-3282(90)90057-6 2192750

[B100] Nakashima T. Hayashi M. Fukunaga T. Kurata K. Oh-hora M. Feng J. Q. (2011). Evidence for osteocyte regulation of bone homeostasis through RANKL expression. Nat. Med. 17, 1231–1234. 10.1038/nm.2452 21909105

[B101] Osório J. (2015). Bone. Osteocyte-specific activation of the canonical Wnt-β catenin pathway stimulates bone formation. Nat. Rev. Endocrinol. 11, 192. 10.1038/nrendo.2015.11 25645703

[B102] Ozawa H. Imamura K. Abe E. Takahashi N. Hiraide T. Shibasaki Y. (1990). Effect of a continuously applied compressive pressure on mouse osteoblast-like cells (MC3T3-E1) *in vitro* . J. Cell. Physiol. 142, 177–185. 10.1002/jcp.1041420122 2298821

[B103] Pacheco-Costa R. Davis H. M. Sorenson C. Hon M. C. Hassan I. Reginato R. D. (2015). Defective cancellous bone structure and abnormal response to PTH in cortical bone of mice lacking Cx43 cytoplasmic C-terminus domain. Bone 81, 632–643. 10.1016/j.bone.2015.09.011 26409319 PMC4640960

[B104] Paznekas W. A. Boyadjiev S. A. Shapiro R. E. Daniels O. Wollnik B. Keegan C. E. (2003). Connexin 43 (GJA1) mutations cause the pleiotropic phenotype of oculodentodigital dysplasia. Am. J. Hum. Genet. 72, 408–418. 10.1086/346090 12457340 PMC379233

[B105] Penuela S. Gehi R. Laird D. W. (2013). The biochemistry and function of pannexin channels. Biochim. Biophys. Acta 1828, 15–22. 10.1016/j.bbamem.2012.01.017 22305965

[B106] Plotkin L. I. Davis H. M. Cisterna B. A. Sáez J. C. (2017). Connexins and pannexins in bone and skeletal muscle. Curr. Osteoporos. Rep. 15, 326–334. 10.1007/s11914-017-0374-z 28647887 PMC5544010

[B107] Plotkin L. I. Mathov I. Aguirre J. I. Parfitt A. M. Manolagas S. C. Bellido T. (2005). Mechanical stimulation prevents osteocyte apoptosis: Requirement of integrins, src kinases, and ERKs. Am. J. Physiol. Cell. Physiol. 289, C633–C643. 10.1152/ajpcell.00278.2004 15872009

[B108] Riquelme M. A. Burra S. Kar R. Lampe P. D. Jiang J. X. (2015). Mitogen-activated protein kinase (MAPK) activated by prostaglandin E2 phosphorylates connexin 43 and closes osteocytic hemichannels in response to continuous flow shear stress. J. Biol. Chem. 290, 28321–28328. 10.1074/jbc.M115.683417 26442583 PMC4653687

[B109] Riquelme M. A. Gu S. Hua R. Jiang J. X. (2021). Mechanotransduction via the coordinated actions of integrins, PI3K signaling and Connexin hemichannels. Bone Res. 9, 8. 10.1038/s41413-020-00126-w 33531460 PMC7854719

[B110] Riquelme M. A. Kar R. Gu S. Jiang J. X. (2013). Antibodies targeting extracellular domain of connexins for studies of hemichannels. Neuropharmacology 75, 525–532. 10.1016/j.neuropharm.2013.02.021 23499293 PMC3718874

[B111] Romanello M. D'Andrea P. (2001). Dual mechanism of intercellular communication in HOBIT osteoblastic cells: A role for gap-junctional hemichannels. J. Bone Min. Res. 16, 1465–1476. 10.1359/jbmr.2001.16.8.1465 11499869

[B112] Romanello M. Pani B. Bicego M. D'Andrea P. (2001). Mechanically induced ATP release from human osteoblastic cells. Biochem. Biophys. Res. Commun. 289, 1275–1281. 10.1006/bbrc.2001.6124 11741333

[B113] Romanello M. Veronesi V. D'Andrea P. (2003). Mechanosensitivity and intercellular communication in HOBIT osteoblastic cells: A possible role for gap junction hemichannels. Biorheology 40, 119–121. 12454395

[B114] Sample S. J. Behan M. Smith L. Oldenhoff W. E. Markel M. D. Kalscheur V. L. (2008). Functional adaptation to loading of a single bone is neuronally regulated and involves multiple bones. J. Bone Min. Res. 23, 1372–1381. 10.1359/jbmr.080407 PMC2586809 18410233

[B115] Saunders M. M. You J. Trosko J. E. Yamasaki H. Li Z. Donahue H. J. (2001). Gap junctions and fluid flow response in MC3T3-E1 cells. Am. J. Physiol. Cell. Physiol. 281, c1917–c1925. 10.1152/ajpcell.2001.281.6.C1917 11698250

[B116] Saunders M. M. You J. Zhou Z. Li Z. Yellowley C. E. Kunze E. L. (2003). Fluid flow-induced prostaglandin E2 response of osteoblastic ROS 17/2.8 cells is gap junction-mediated and independent of cytosolic calcium. Bone 32, 350–356. 10.1016/s8756-3282(03)00025-5 12689677

[B117] Sawakami K. Robling A. G. Ai M. Pitner N. D. Liu D. Warden S. J. (2006). The Wnt co-receptor LRP5 is essential for skeletal mechanotransduction but not for the anabolic bone response to parathyroid hormone treatment. J. Biol. Chem. 281, 23698–23711. 10.1074/jbc.m601000200 16790443

[B118] Shen H. Grimston S. Civitelli R. Thomopoulos S. (2015). Deletion of connexin43 in osteoblasts/osteocytes leads to impaired muscle formation in mice. J. Bone Min. Res. 30, 596–605. 10.1002/jbmr.2389 PMC4444057 25348938

[B119] Siller-Jackson A. J. Burra S. Gu S. Xia X. Bonewald L. F. Sprague E. (2008). Adaptation of connexin 43-hemichannel prostaglandin release to mechanical loading. J. Biol. Chem. 283, 26374–26382. 10.1074/jbc.M803136200 18676366 PMC2546557

[B120] Spector E. R. Smith S. M. Sibonga J. D. (2009). Skeletal effects of long-duration head-down bed rest. Aviat. Space Environ. Med. 80, A23–A28. 10.3357/asem.br02.2009 19476166

[B121] Sugiatno E. Samsudin A. R. Ibrahim M. F. Sosroseno W. (2006). The effect of nitric oxide on the production of prostaglandin E2 by hydroxyapatite-stimulated a human osteoblast (HOS) cell line. Biomed. Pharmacother. 60, 147–151. 10.1016/j.biopha.2006.02.008 16581222

[B122] Thi M. M. Islam S. Suadicani S. O. Spray D. C. (2012). Connexin43 and pannexin1 channels in osteoblasts: Who is the "hemichannel. J. Membr. Biol. 245, 401–409. 10.1007/s00232-012-9462-2 22797941 PMC3427001

[B123] Thi M. M. Urban-Maldonado M. Spray D. C. Suadicani S. O. (2010). Characterization of hTERT-immortalized osteoblast cell lines generated from wild-type and connexin43-null mouse calvaria. Am. J. Physiol. Cell. Physiol. 299, C994–C1006. 10.1152/ajpcell.00544.2009 20686067 PMC2980299

[B124] Thorsen K. Kristoffersson A. O. Lerner U. H. Lorentzon R. P. (1996). *In situ* microdialysis in bone tissue. Stimulation of prostaglandin E2 release by weight-bearing mechanical loading. J. Clin. Invest. 98, 2446–2449. 10.1172/JCI119061 8958205 PMC507700

[B125] Tian X. Y. Zhang Q. Zhao R. Setterberg R. B. Zeng Q. Q. Ma Y. F. (2007). Continuous infusion of PGE2 is catabolic with a negative bone balance on both cancellous and cortical bone in rats. J. Musculoskelet. Neuronal Interact. 7, 372–381. 18094515

[B126] Tu X. Delgado-Calle J. Condon K. W. Maycas M. Zhang H. Carlesso N. (2015). Osteocytes mediate the anabolic actions of canonical Wnt/β-catenin signaling in bone. Proc. Natl. Acad. Sci. U. S. A. 112, E478–E486. 10.1073/pnas.1409857112 25605937 PMC4321271

[B127] Ueno K. Haba T. Woodbury D. Price P. Anderson R. Jee W. S. (1985). The effects of prostaglandin E2 in rapidly growing rats: Depressed longitudinal and radial growth and increased metaphyseal hard tissue mass. Bone 6, 79–86. 10.1016/8756-3282(85)90311-4 3860213

[B128] Vesely P. Boyde A. Jones S. J. (1992). Behaviour of osteoclasts *in vitro*: Contact behaviour of osteoclasts with osteoblast-like cells and networking of osteoclasts for 3D orientation. J. Anat. 181, 277–291. 1295866 PMC1259723

[B129] Wang Y. McNamara L. M. Schaffler M. B. Weinbaum S. (2007). A model for the role of integrins in flow induced mechanotransduction in osteocytes. Proc. Natl. Acad. Sci. U. S. A. 104, 15941–15946. 10.1073/pnas.0707246104 17895377 PMC2000405

[B130] Warden S. J. Fuchs R. K. Castillo A. B. Nelson I. R. Turner C. H. (2007). Exercise when young provides lifelong benefits to bone structure and strength. J. Bone Min. Res. 22, 251–259. 10.1359/jbmr.061107 17129172

[B131] Watkins M. Grimston S. K. Norris J. Y. Guillotin B. Shaw A. Beniash E. (2011). Osteoblast connexin43 modulates skeletal architecture by regulating both arms of bone remodeling. Mol. Biol. Cell. 22, 1240–1251. 10.1091/mbc.E10-07-0571 21346198 PMC3078079

[B132] Watkins M. P. Norris J. Y. Grimston S. K. Zhang X. Phipps R. J. Ebetino F. H. (2012). Bisphosphonates improve trabecular bone mass and normalize cortical thickness in ovariectomized, osteoblast connexin43 deficient mice. Bone 51, 787–794. 10.1016/j.bone.2012.06.018 22750450 PMC3432742

[B133] Welch R. D. Johnston C. E. 2nd Waldron M. J. Poteet B. (1993). Intraosseous infusion of prostaglandin E2 in the caprine tibia. J. Orthop. Res. 11, 110–121. 10.1002/jor.1100110113 8423513

[B134] Wijenayaka A. R. Kogawa M. Lim H. P. Bonewald L. F. Findlay D. M. Atkins G. J. (2011). Sclerostin stimulates osteocyte support of osteoclast activity by a RANKL-dependent pathway. PLoS One 6, e25900. 10.1371/journal.pone.0025900 21991382 PMC3186800

[B135] Woodward D. F. Jones R. L. Narumiya S. (2011). International union of basic and clinical pharmacology. LXXXIII: Classification of prostanoid receptors, updating 15 years of progress. Pharmacol. Rev. 63, 471–538. 10.1124/pr.110.003517 21752876

[B136] Xia X. Batra N. Shi Q. Bonewald L. F. Sprague E. Jiang J. X. (2010). Prostaglandin promotion of osteocyte gap junction function through transcrip-tional regulation of connexin 43 by glycogen synthase kinase 3/βcatenin signaling. Mol. Cell. Biol. 30, 206–219. 10.1128/mcb.01844-08 19841066 PMC2798309

[B137] Xiong J. Onal M. Jilka R. L. Weinstein R. S. Manolagas S. C. O'Brien C. A. (2011). Matrix-embedded cells control osteoclast formation. Nat. Med. 17, 1235–1241. 10.1038/nm.2448 21909103 PMC3192296

[B138] Xu H. Gu S. Riquelme M. A. Burra S. Callaway D. Cheng H. (2015). Connexin 43 channels are essential for normal bone structure and osteocyte viability. J. Bone Min. Res. 30, 436–448. 10.1002/jbmr.2374 PMC4333056 25270829

[B139] Xu H. Liu R. Ning D. Zhang J. Yang R. Riquelme M. A. (2017). Biological responses of osteocytic connexin 43 hemichannels to simulated microgravity. J. Orthop. Res. 35, 1195–1202. 10.1002/jor.23224 26945892 PMC5726230

[B140] Yang R. S. Liu T. K. Lin-Shiau S. Y. (1993). Increased bone growth by local prostaglandin E2 in rats. Calcif. Tissue Int. 52, 57–61. 10.1007/BF00675627 8453506

[B149] Yang X. Zeng H. Wang L. Luo S. Zhou Y. (2022). Activation of Piezo1 downregulates renin in juxtaglomerular cells and contributes to blood pressure homeostasis. Cell. Biosci. 12, 197. 10.1186/s13578-022-00931-2 36471394 PMC9720979

[B141] Yellowley C. E. Li Z. Zhou Z. Jacobs C. R. Donahue H. J. (2000). Functional gap junctions between osteocytic and osteoblastic cells. J. Bone Min. Res. 15, 209–217. 10.1359/jbmr.2000.15.2.209 10703922

[B142] Zeng Q. Wang Y. Gao J. Yan Z. Li Z. Zou X. (2019). miR-29b-3p regulated osteoblast differentiation via regulating IGF-1 secretion of mechanically stimulated osteocytes. Cell. Mol. Biol. Lett. 24, 11. 10.1186/s11658-019-0136-2 30915127 PMC6416934

[B143] Zeng Y. Riquelme M. A. Hua R. Zhang J. Acosta F. M. Gu S. (2022). Mechanosensitive piezo1 calcium channel activates connexin 43 hemichannels through PI3K signaling pathway in bone. Cell. Biosci. 12, 191. 10.1186/s13578-022-00929-w 36457052 PMC9716748

[B144] Zhang Y. Paul E. M. Sathyendra V. Davison A. Sharkey N. Bronson S. (2011). Enhanced osteoclastic resorption and responsiveness to mechanical load in gap junction deficient bone. PLoS One 6, e23516. 10.1371/journal.pone.0023516 21897843 PMC3163577

[B145] Zhao D. Hua R. Riquelme M. A. Cheng H. Guda T. Xu H. (2022b). Osteocytes regulate bone anabolic response to mechanical loading in male mice via activation of integrin α5. Bone Res. 10, 49. 10.1038/s41413-022-00222-z 35851577 PMC9293884

[B146] Zhao D. Liu R. Li G. Chen M. Shang P. Yang H. (2020). Connexin 43 channels in osteocytes regulate bone responses to mechanical unloading. Front. Physiol. 11, 299. 10.3389/fphys.2020.00299 32296345 PMC7137730

[B147] Zhao D. Riquelme M. A. Guda T. Tu C. Xu H. Gu S. (2022a). Connexin hemichannels with prostaglandin release in anabolic function of bone to mechanical loading. eLife 11, e74365. 10.7554/eLife.74365 35132953 PMC8824479

